# Perspective: Identification of genetic variants associated with dopaminergic compensatory mechanisms in early Parkinson's disease

**DOI:** 10.3389/fnins.2013.00052

**Published:** 2013-04-15

**Authors:** Lior Greenbaum, Mordechai Lorberboym, Eldad Melamed, Amihai Rigbi, Yael Barhum, Yoav Kohn, Alexander Khlebtovsky, Bernard Lerer, Ruth Djaldetti

**Affiliations:** ^1^Biological Psychiatry Laboratory, Department of Psychiatry, Hadassah - Hebrew University Medical CenterJerusalem, Israel; ^2^Department of Nuclear Medicine, Edith Wolfson Medical CenterHolon, Israel; ^3^Sackler Faculty of Medicine, Tel Aviv UniversityTel Aviv, Israel; ^4^Department of Neurology, Rabin Medical CenterPetah Tiqva, Israel; ^5^Laboratory of Neuroscience, Felsenstein Research CenterPetah Tiqva, Israel

**Keywords:** Parkinson's disease, compensatory mechanisms, FP-CIT SPECT, tyrosine hydroxylase, neuroimaging genetics

## Abstract

Parkinson's disease (PD) is slowly progressive, and heterogeneity of its severity among individuals may be due to endogenous mechanisms that counterbalance the striatal dopamine loss. In this perspective paper, we introduce a neuroimaging-genetic approach to identify genetic variants, which may contribute to this compensation. First, we briefly review current known potential compensatory mechanisms for premotor and early disease PD, located in the striatum and other brain regions. Then, we claim that a mismatch between mild symptomatic disease, manifested by low motor score on the Unified PD Rating Scale (UPDRS), and extensive Nigro-Striatal (NS) degeneration, manifested by reduced uptake of [^123^I]FP-CIT, is indicative of compensatory processes. If genetic variants are associated with the severity of motor symptoms, while the level of striatal terminals degeneration measured by ligand uptake is taken into account and controlled in the analysis, then these variants may be involved in functional compensatory mechanisms for striatal dopamine deficit. To demonstrate feasibility of this approach, we performed a small “proof of concept” study (candidate gene design) in a sample of 28 Jewish PD patients, and preliminary results are presented.

## Introduction

Parkinson's disease (PD) is characterized by progressive degeneration of dopaminergic neurons in the substantia nigra, causing depletion in striatal dopamine level, variable clinical expressions, and a slowly deteriorating course (Dauer and Przedborski, 2003; Obeso et al., 2010). In general, there is an association between disease severity and disease duration, but the rate of disease progression is variable among patients. Thus far, there are no biological markers that can predict rate of disease progression in an individual patient (Kuriakose and Stoessl, 2010).

The inter-individual difference in severity of motor symptoms during early and mid duration of PD may be attributed to existence or lack of compensatory mechanisms for dopamine loss (Obeso et al., 2004). Thus far, research of compensatory mechanisms has mostly concentrated on the transition between pre-motor stage and the emergence of the motor symptoms (Bezard et al., 2003). However, it is also plausible that compensatory mechanisms play a role in the motor symptomatic period, thus manipulating disease severity and progression (Brotchie and Fitzer-Attas, 2009). These functional mechanisms may counterbalance dopamine loss in early PD, attenuating the severity and progression of motor symptoms (Biju and de la Fuente-Fernández, 2009). Identification of such mechanisms is of major scientific and clinical importance.

Herein we introduce an original approach to discover genetic variants contribution to endogenous compensatory mechanisms in early PD, achieved by combining clinical, genetic and neuroimaging data. Our hypothesis is that a mismatch between mild symptomatic disease, manifested by low motor score on the Unified PD Rating Scale (UPDRS), and extensive Nigro-Striatal (NS) degeneration, manifested by reduced uptake of [^123^I]FP-CIT, is indicative of compensatory processes. Genetic variations probably predispose some individuals to better compensatory potential for the striatal dopamine loss. We believe that this type of systematic research will offer initial tools required to identify these variants, and to trace genes that may be involved in compensatory mechanisms.

This perspective paper briefly reviews several of the currently known compensatory mechanisms in PD. Then, we present our algorithm and report initial findings of a small “proof of concept” study for identification of genetic compensatory mechanisms in early PD. Naturally, the small sample size used in this report is underpowered, but the goal in this context is to present the new method and its possible applicability, and not to reach definitive conclusions.

## Compensatory mechanisms in presymptomatic and early PD disease

It is well established that parkinsonian signs appear when 70–80% of the dopaminergic neurons are already lost (Bernheimer et al., 1973; Lee et al., 2000; Perez et al., 2008; de la Fuente-Fernández et al., 2011). The postmortem analysis of parkinsonian brains indicates that extensive loss of dopamine in the putamen and in the caudate nucleus can still be accompanied by only minor clinical symptoms (Bernheimer et al., 1973; Agid, 1991). According to Braak's pathological staging of PD, the process begins in the dorsal nucleus of the vagus and the olfactory bulb, reaching the substantia nigra only in the third pathological stage (Braak et al., 2003). This explains the appearance of autonomic and other non-motor symptoms before the appearance of the motor ones. Another explanation might be that intra- and extrastriatal compensatory mechanisms are responsible for the late manifestation of motor symptoms (Brotchie and Fitzer-Attas, 2009). After the appearance of the first motor manifestations, degeneration of dopaminergic neurons continues, causing further decline in motor performance. However, the variability of disease progression among patients is high, indicating that compensatory mechanisms play a role also in this stage. These mechanisms may be related to genetic predisposition, but also to environmental ones [e.g., consumption of nicotine and caffeine—which have a neuroprotective effect (Sugita et al., 2001; Quik et al., 2007; Prediger, 2010)]. Demographic factors, like age at disease onset, also influence compensatory potential, in favor of younger age (de la Fuente-Fernández et al., 2011).

### Striatal compensatory mechanisms that aim to maximize the effect of the remaining dopamine in the striatum, pre- and postsynapticlly

#### Upregulation of enzymes involved in dopamine metabolism

Several studies have shown upregulation of tyrosine hydroxylase (TH) and aromatic acid decarboxylase (AADC) in brains of animal models of PD (Zigmond et al., 1984, 1990; Lee et al., 2000), leading to increased dopamine synthesis in the residual neurons. Longitudinal PET studies demonstrated that upregulation of the dopamine synthesis declines as PD progresses (Nandhagopal et al., 2011). Intranigral injections of 6-hydroxydopamine accelerated the rates of dopamine synthesis and release by the surviving nigrostriatal neurons by increasing TH activity (Melamed et al., 1982). Evidence of increased ratio of dopamine metabolites to dopamine supports this theory (Hefti et al., 1985; Elsworth et al., 2000; Bezard et al., 2001b). However, it has been shown that the remaining dopaminergic neurons in the substantia nigra in patients with PD exhibit lower rather than higher TH mRNA expression, rendering this theory less plausible (Javoy-Agid et al., 1990).

#### Upregulation of D2 dopamine receptor expression on striatal neurons

Development of striatal dopamine receptor super-sensitivity may augment responsiveness to remaining dopamine. This phenomena is mainly related to raised level of dopamine D2 receptors. Several human and primate studies have demonstrated postsynaptic supersensitivity, mediated by increase of D2 receptors binding (Creese et al., 1977; Lee et al., 1978; Falardeau et al., 1988; Todd et al., 1996; Bezard et al., 2001b). This means that depletion of dopaminergic nigrostriatal output leads to increased D2 receptors number (on medium spiny neurons), to compensate the underactivation of the downstream transduction pathways.

In contrast to D2 receptors, most studies do not support super-sensitization of D1 receptors following dopamine depletion, in terms of increased number and affinity (Shinotoh et al., 1993; Hurley et al., 2001; Stoessl et al., 2011), albeit reports of increased D1 receptors recruitment to the plasma membrane of medium spiny neurons (Guigoni et al., 2007). Principally, Increased dopamine receptor expression in the striatum may attenuate the functional imbalance in PD, characterized by overactivation of the indirect pathway (see below), and underactivation of the direct pathway (Jenner, 2008). Overexpression of D2 receptors enhances the inhibitory effect of the remaining dopamine in the striatum on the activity of the indirect pathway, while overexpression of D1 receptors might facilitate the activation of the direct pathway—and delay manifestations of PD motor symptoms.

Interestingly, alternations of cerebral blood volume in the striatum are induced by activation or blockade of various dopamine receptor subtypes (Choi et al., 2006; Sánchez-Pernaute et al., 2007; Chen et al., 2012). Changes in vascular tone may occur due to activation of receptors expressed on astrocytes, affecting basal ganglia response to various stimuli and have a compensatory role.

#### Down-regulation of the dopamine transporter (DAT)

In PD, down-regulation of DAT which modulates concentration of synaptic dopamine should cause decreased dopamine reuptake. This results in higher concentration of dopamine in the synapse (Lee et al., 2000; Sossi et al., 2007). Existence of this potential mechanism is supported by findings of reduced DAT level per surviving dopaminergic neuron in PD (Uhl et al., 1994).

#### Structural mechanisms

Other putative compensatory mechanisms are structural, involving increase in the number of intrinsic striatal TH interneurons. This neuron population, positioned within the striatum itself, is able to produce dopamine (Porritt et al., 2000; Huot et al., 2007). It is still unclear whether these TH neurons growth is a production of new neurons (neurogenesis), or represents striatal mature neurons which adopt the TH phenotype and the ability to produce dopamine (phenotypic shift), acting as a compensatory mechanism (Tandé et al., 2006; Huot et al., 2007). This process might be influenced by several factors, such as presence and concentrations of required growth factors (e.g., BDNF, GDNF) (Du and Iacovitti, 1995). In addition, growth factors may support generation of dopaminergic terminal on the surviving NS neurons, as well as promote other compensatory structural mechanisms within the striatum (Rosenblad et al., 1998; Weinreb et al., 2007).

#### Transaxonal regulation

In a recent study, ablation of the TH gene in mice revealed that TH protein levels in the axon terminals are regulated differently from that in the soma, and that tissue dopamine levels are under trans-axonal compensatory regulation: The reduction of dopamine in some axons induces enhanced dopamine synthesis in others (Tokuoka et al., 2011). Trans-axonal compensation may be mediated by phsophorylation of TH, decreased dopamine reuptake or by neurotrophic factors.

### Extra striatal compensatory mechanisms

The indirect pathway projects from the striatum to the Globus Pallidus externa (GPe), and inhibits it via GABAergic transmission. Reduction of GPe inhibition by the indirect pathway can serve as a compensatory mechanism to dopamine depletion, and reduce PD symptoms severity (Brotchie and Fitzer-Attas, 2009; Black et al., 2010).

Several mechanisms were suggested to reduce activity of the indirect pathway, in addition to dopaminergic ones. Decreased glutamatergic excitation probably has a compensatory potential, and might be mediated by the endocannabinoid system. Increased endocannabinoid levels (achieved by lower degradation through the FAAH enzyme) reduces striatal glutamate release (Gubellini et al., 2002; Pisani et al., 2005; van der Stelt et al., 2005), and therefore attenuate the indirect pathway activity. Other mechanisms focus on enkephalinergic signaling, which may reduce GABA release by the terminals of the indirect pathway, and decrease its inhibitory influence on the GPe (Asselin et al., 1994; Herrero et al., 1995). There is evidence for upregulation of enkephalin transmission in PD before the manifestation of motor symptoms (Dacko and Schneider, 1991; Bezard et al., 2001c), rendering it an attractive potential compensatory mechanism, balancing the overactive inhibitory GABA input to GPe.

In animal models, overactivity of the subthalamic nucleus (STN) and globus pallidum interna (GPi) before the appearance of overt PD symptoms has been demonstrated (Vila et al., 2000; Obeso and Schapira, 2009). However, it is not clear if these changes are indeed compensatory by their character.

Deep brain stimulation (DBS) to extra-striatal brain regions (e.g., Subthalamic nucleus, STN) is an established therapy for advanced PD, inhibits neuronal activity at stimulation site. Interestingly, several studies suggested that STN DBS may also facilitate induction of compensatory mechanisms and networks, relevant to movement control (Johnson et al., 2008).

### Cortical and cerebellar level

This category of compensatory mechanisms is located in brain regions and networks involved in motor system control, outside the basal ganglia (Bezard et al., 2001a; Palmer et al., 2009; Appel-Cresswell et al., 2010). These regions might adapt themselves to dopamine depletion state in the straitum, balancing the impaired basal ganglia function (Palmer et al., 2009). Increased activity of the Supplementary Motor Area (SMA) and the right dorsal pre-motor cortex was observed in healthy individual carrying the PINK1 mutation (interpreted as compensatory to the latent NS dysfunction) (van Nuenen et al., 2009). There is some support in animal models that in addition to NS degeneration, a decreased metabolic activity of the SMA is required to the appearance of PD motor symptoms (Rascol et al., 1992, 1998). Therefore, increased SMA activity may delay overt manifestation of motor symptoms.

At the mechanistic level, reduction of regional metabolism in PD is consistently reported in several cortical areas (Stoessl et al., 2011). Alternation of cerebral blood flow and volume, as well as in glucose metabolism may be relevant to compensatory strategies (Appel-Cresswell et al., 2010). In addition, compensatory and beneficial changes include increased activation of neuronal networks and recruitment of novels ones (Palmer et al., 2009), as well as alternation of connectivity patterns between brain regions (Palmer et al., 2010). For example, compensatory role for altered dorsal pre-motor cortex activity among PD patients was found in a visual region in the occipito-temporal cortex (van Nuenen et al., 2012), as well for increased motor cortical plasticity and reorganization of the sensorimotor cortex (Kojovic et al., 2012).

Moreover, increased activity of the cerebellum and its projections may compensate for basal ganglia impaired function (Cerasa et al., 2006). In a neuroimaging study of motor task performance, higher activity of the cerebellum compensated for reduced putaminal activity (Yu et al., 2007). Cortico-cerebellar circuits are assumed to play a compensatory role, but findings should be interpreted with caution, since these changes may also be related to PD pathology itself (Martinu and Monchi, 2012).

## A new approach for identification of genetic compensatory mechanisms

Our hypothesis focuses on the possible influence of genetic variants on severity of symptoms in early PD. We became aware to patients with marked decrease of [^123^I]FP-CIT uptake in the striatum at early stage of the disease, while their clinical phenotype was compatible with a mild disease. We assume that a mismatch between dopamine binding and symptom severity may be indicative of compensatory mechanisms that act in the early motor stage of the disease.

The basic hypothesis for our model is that if genetic variants are associated with the severity of motor symptoms, while the level of striatal terminals degeneration measured by striatal [^123^I]FP-CIT uptake is taken into account and controlled in the analysis, then these variants may be involved in functional compensatory mechanisms for the dopamine deficit in the striatum. Our algorithm is based on a statistical analysis which combines three parameters: clinical, genetic and neuroimaging data.

Following selection of Single Nucleotide Polymorphisms (SNPs) of interest and genotyping them in a suitable sample of PD patients, multivariate linear regression model is implemented to predict UPDRS score as a function of selected SNPs together with relevant covariates (gender, age, disease duration). The UPDRS score (indicator of motor severity evaluated in unmedicated patients or if already treated with dopaminergic agents, after a “wash-out” period) is the dependent variable in this association study, and the SNP genotype is the independent variable (linear regression). We first force enter [^123^I]FP-CIT uptake values as a covariate that controls for degree of striatal terminals degeneration. Then, a second block is entered into the model using stepwise method, contains a single SNP. Thus, this SNP potential unique contribution to the UPDRS variance could be examined. This step is performed separately for each of the studied SNPs of interests.

To demonstrate the feasibility of this algorithm, we have performed a small “proof of concept” candidate gene study. The study group consisted of 28 Israeli Jewish patients with early idiopathic PD who had not yet been treated with any anti-PD drugs. The study was approved by the Ethics Committee of Rabin Medical Center, and all patients signed an informed consent form. All patients underwent [^123^I]-FP-CIT SPECT within few weeks from assessment of severity of the motor symptoms by the UPDRS motor sub-score. For SPECT imaging and analysis, see Appendix Methods.

Tagging SNPs were selected in 4 candidate genes participating in dopamine metabolism pathway: tyrosine hydroxylase (TH), catechol-O-methyltransferase (COMT), and monoamine oxidase (MAO) A and B. The selection was performed on the basis of the international HapMap project CEU group data, using the tagger option of the Haploview program, version 4.1 (Barrett et al., 2005). Criteria for inclusion were minor allele frequency of >5% and minimum *r*^2^ threshold of 0.8. In total, twenty-six SNPs were successfully genotyped.

The first stage was to evaluate univariate associations between these SNPs and the UPDRS score. Table A1 shows that only the rs6356 SNP in the TH gene (encoding for amino acid Val to Met substitution at position 81) showed a nominal significant association with the UPDRS score (*p* = 0.007).

**Figure A1:**
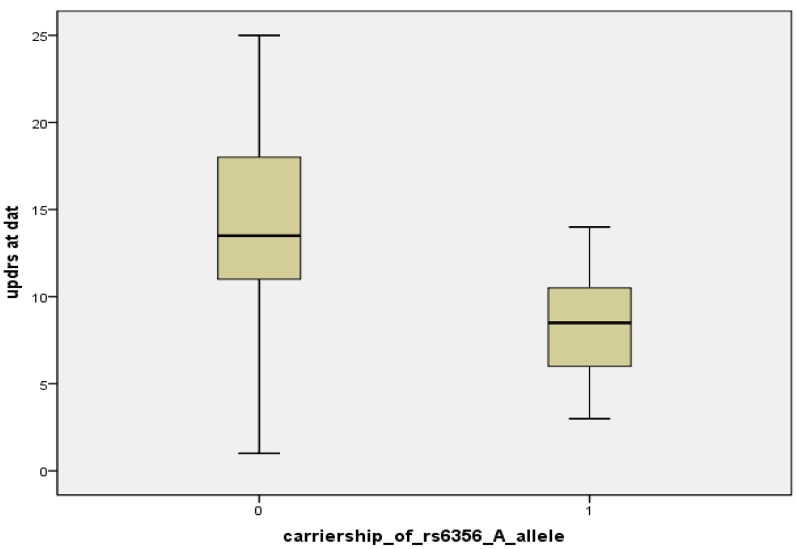
**Boxplot of UPDRS scores at DAT scan, classified by carriership of the rs6356 “A” allele [“0” means non-carriers of “A” allele (*n* = 8), “1” means carriers “A” allele (AA or AG genotype) (*n* = 20)]**.

Table 1 shows the clinical characteristics and FP-CIT uptake data of the whole sample and of the carriers/non-carriers of the rs6356 minor allele. Twenty patients were carriers of the rs6356 AA/AG genotypes, and 8 were carriers of the major allele “G” homozygous genotype (GG). There was a trend for shorter disease duration among the carriers of the “A” allele compared to homozygotes for the “G” allele (*p* = 0.06). The mean UPDRS score was significantly lower in the AA/AG genotype carriers than the GG homozygotes (*p* = 0.007) despite the lack of significant differences in [^123^]FP-CIT uptake in the contralateral (to the more affected side of the body) putamen, caudate, or mean putaminal uptake between these groups (Figure A1).

**Table 1:** **Demographic, clinical, radiotracer uptake values, and descriptive statistics of the whole sample and of carriers and non-carriers of the rs6365 SNP**.

	**Whole sample**	**rs6356: Carriers of A allele (AA + AG)**	**rs6356: Non-carriers of A allele (GG)**	***P*-value (*t*-test)**
No. of patients	28	20	8	
Age (years), mean ± SD	65.7 ± 7.6	65.6 ± 7.4	65.8 ± 8.8	0.94
Males, n (%)	20 (71%)	15 (75%)	5 (62.5%)	0.41^*^
Disease duration at SPECT (years), mean ± SD	1.73 ± 1.6	1.4 ± 1.3	2.6 ± 0	0.06
UPDRS at SPECT, mean ± SD	9.85 ± 5.17	8.25 ± 3.22	13.8 ± 7.0	**0.007**
Radiotracer absorption				
Contralateral putamen	1.42 ± 0.6	1.39 ± 0.53	1.5 ± 0.77	0.66
Contralateral caudate	2.66 ± 0.81	2.62 ± 0.76	2.76 ± 0.98	0.69
Mean putamen (ipsi- and contralateral)	1.62 ± 0.65	1.57 ± 0.58	1.73 ± 0.84	0.44

* *Chi-square test. All other variables were analyzed by t-test. Bold value indicates *p* < 0.05*.

The second stage examined the effect of rs6356 on disease severity using stepwise linear regression. The SPECT data and disease duration (block 1, control variables) were force-entered first, and then the gene variant was added using the stepwise method (block 2). The two control variables, by themselves, explained 31% of the inter-individual variance in the UPDRS scores (*R*^2^ = 0.31, *p* = 0.009) (Table 2). After the second block (containing only the rs6356 SNP) was entered, the model yielded a total *R*^2^ of 0.45. This means that when the duration of PD and the level of ligand uptake were held constant, the contribution of the rs6356 “A” allele (AA and AG genotypes) was statistically significant (*p* = 0.01), with rs6356 explaining about one-third (*R*^2^ = 0.14) of the total variance in UPDRS scores. Adding the genetic variable to a model that included non-genetic predictors of PD severity may increase the percentage of variance explained by the model (Table 2). Similar and consistent results were achieved with values of the contralateral caudate and mean putamen uptake (Table A2).

**Table 2:** **Summary of the linear regression model predicting UPDRS score by model; Block 2 was entered in a stepwise manner**.

**Block**	**Variable**	***B***	***SE***	β	***t***	***p*-value**	**Cumulative ***R***^**2**^**
**LINEAR REGRESSION MODEL PREDICTING UPDRS BY TRACER UPTAKE IN THE CONTRALATERAL PUTAMEN**							
	Constant	15.04	3.36		4.47	<0.001	
1	Duration of PD (months)	0.937	0.563	0.291	1.664	0.937	
	Absorption level in contralateral putamen	−2.4	1.42	−0.27	−1.69	0.1	0.31 (*p* = 0.009)
2	rs6356 (carriership of A allele)	−4.72	1.86	−0.42	−2.53	0.01	0.45 (*p* = 0.002)

In this example, the Val81Met polymorphism (rs6356) in the TH gene influences disease severity in our small sample of patients with early PD. The contribution of this genetic variant to the prediction of UPDRS score is adjunctive to the combined effect of variables of FP-CIT uptake and disease duration, as demonstrated by linear regression analysis. In other words, the genetic variant has an effect on motor symptoms severity in PD when level of NS degeneration level and disease duration are held constant, and therefore may be involved in compensatory mechanisms of the disease.

The results of this “proof of concept” study should be interpreted with caution, and may actually represent false-positive findings, due to the small sample size and the burden of multiple testing. TH association does not withstand correction for multiple testing (required *p*-value of 0.002, according to Benofferoni correction). In general, the best strategy to confirm results of genetic association studies and to differentiate true positive from false negative results, is to perform a replication study in an independent sample. Unfortunately, we do not have access to a validation sample, which contains the required data for this analysis. It should be recognized that our sample is unique, as all participants were of Jewish ancestry (reduced genetic heterogeneity), and naïve to anti-Parkinson medications at the time of clinical assessment and SPECT scanning. If drug-treated patients are included in future samples, then motor assessment should be performed following an acceptable “wash-out” period.

Nevertheless, our goal here was to present the novel method in a “hypothesis generating” manner. Larger and well powered samples are warranted to confirm (or deny) the current preliminary results, and to discover additional potential genetic variants associated with compensatory potential in PD.

## Future direction in neuroimaging-genetic studies for PD's compensatory mechanisms

In contrast to the candidate gene approach, implementing the hypothesis free GWAS, will enable to identify other genes, not restricted to the “usual suspects” of dopamine homeostasis in the striatum but also unexpected ones, advancing the field beyond current knowledge. These may include, for example, genes and pathways involved in novel mechanisms of attenuating the indirect pathway activity, or affecting regions and network in the cortex and cerebellum. It should be acknowledged, however, that the current analytic approach is in line with the “common disease-common variant” hypothesis, resuming that large number of common genetic variants account for the genetic contribution to the phenotype, and each SNP has a small size effect. However, low frequency and even rare variants (with large effect size and variable penetrance) may also play role in genetic contribution to compensatory mechanisms in PD. To systematically evaluate the compensatory potential of low frequency/rare variants such as these, very large samples are required, incorporating methods such as whole exome/genome sequencing.

Beside valuable insight to development of PD modifying drugs that slow rate of disease progression, identification of genetic variants involved in endogenous compensatory mechanisms can lead to “individualized medicine.” A priori identification of patients carrying variants that increase the compensatory potential may have impact on therapeutic decisions. This can also bear implications for patients' stratification in clinical trials, evaluating efficacy of novel drugs.

Our genomic imaging model, with appropriate adaptation, might be applicable not only for prediction of motor severity in PD but also to other non-motor symptoms such as dementia. Also, it can be used in other neurodegenerative diseases. For example, improvement of imaging strategies in Alzheimer disease will enable to find genes and molecular pathways involved in disease progression, in a similar way.

As a final point, one should keep in mind that compensatory mechanisms in PD probably represent complex and different processes in various disease stages, not related only to genetics but also to environmental, demographic and stochastic factors. Careful planning of future studies, taking these important factors into account is required.

### Conflict of interest statement

The authors declare that the research was conducted in the absence of any commercial or financial relationships that could be construed as a potential conflict of interest.

## Appendix methods

### Spect imaging

All patients received potassium iodide orally to block thyroid uptake of free radioactive iodide. A dose of 185 MBq [^123^I]-FP-CIT was injected intravenously, and imaging was performed 3 h later. The SPECT study was performed using a dual-head γ-camera (Helix; Elscint) equipped with a low-energy, high-resolution collimator. A 20% window was centered on the 159-keV photopeak of ^123^I. One hundred twenty frames of 15 s each were acquired into a 128 × 128 image matrix using a circular rotation mode. Transaxial, coronal, and sagittal slices 1 pixel thick were reconstructed using a third-order Metz filter set to 12-mm full width at half maximum. Attenuation was corrected with a constant linear attenuation coefficient of 0.11 cm^−1^.

### Analysis of spect results

For analysis of striatal [^123^I]-FP-CIT binding, the two transaxial slices representing the most intense striatal binding were summed and subjected to qualitative analysis of tracer activity in the striatal regions. For quantitative analysis of tracer uptake, regions of interest (ROIs) were constructed manually with the help of a brain atlas in areas corresponding to the right and left putamen, caudate, and overall striatum. For background evaluation, ROI's were also drawn bilaterally in areas corresponding to the medial occipital lobe. For each ROI, mean counts were measured, and specific [^123^I]-FP-CIT uptake was calculated according to the following formula: *specific [^123^I]-FP-CIT uptake = (mean activity in ROI − mean activity in occipital cortex)/mean activity in occipital cortex*. SPECT analysis was performed by an expert in Nuclear medicine who was blinded to the patients' disease duration.

**Table A1:** **Univariate associations (*t*-tests) of tagging SNPSs and UPDRS scores**.

**Gene**	**SNP**	**Comparison groups (genotypes)**	***t*, *df***	***P*-value**
TH	Rs6356	GG/AA+GA	2.94, 26	**0.007**
	Rs10840489	CC/TT+CT	(−1.95), 25	0.06
	Rs10770141	GG/AA+GA	0.71, 26	0.48
COMT	Rs933271	TT/CC+TC	(−0.38), 26	0.7
	Rs740603	GG/AA+GA	(−0.95), 26	0.34
	Rs16982844^*^	NA	NA	NA
	Rs4646312	TT/CC+TC	0.81, 26	0.42
	Rs4680^^†^^	AA/GG+AG	0.78, 26	0.44
		GG/AA+AG	(−0.27), 26	0.78
	Rs4646316	CC/TT+CT	0.23, 26	0.81
	Rs165774	GG/AA+GA	0.15, 26	0.87
	Rs174696	TT/CC+TC	(−0.03), 26	0.97
	rs9332377	CC/TT+CT	0.60, 26	0.55
	rs737866	TT/CC+TC	(−1.32), 26	0.13
	rs5993883	GG/TT+GT	(−0.16), 26	0.77
MAO-A	rs2235185	CC/TT+CT	0.27, 26	0.78
	rs3027392	GG/AA+AG	(−0.16), 26	0.87
	rs3027399^**^	GG/CC+CG	0.24, 26	0.81
	rs5906957	GG/AA+AG	0.91, 26	0.37
	rs909525	AA/GG+AG	0.7, 26	0.49
MAO-B	rs10521432	GG/AA+AG	1, 26	0.32
	rs1799836	AA/GG+AG	0.82, 25	0.42
	rs2311013^*^	NA	NA	NA
	rs3027448	TT/CC+CT	(−1), 26	0.327
	rs4824562^*^	NA	NA	NA
	rs5905449	GG/AA+AG	1.07, 26	0.29
	rs5905512	AA/GG+AG	0.89, 25	0.38

* *MAF < 0.05*.

** *MAF < 0.1*.

† *The two alleles had equal frequencies (0.5)*.

*Bold value indicates p < 0.05*.

**Table A2:** **Summary of the linear regression model predicting UPDRS score by model; Block 2 was entered in a stepwise manner**.

**Block**	**Variable**	***B***	***SE***	β	***t***	***p*-value**	**Cumulative ***R***^**2**^**
**2A: LINEAR REGRESSION MODEL PREDICTING UPDRS BY TRACER UPTAKE IN THE CONTRALATERAL CAUDATE**							
	Constant	16.69	3.34		4.99	<0.001	
1	Duration of PD (months)	1.143	0.502	0.355	2.274	0.032	
	Absorption level in contralateral caudate	−2.1	0.92	−0.33	−2.27	0.03	0.37 (*p* = 0.003)
2	rs6356 (carriership of A allele)	−4.48	1.75	−0.39	−2.56	0.01	0.5 (*p* = 0.001)
**2B: LINEAR REGRESSION MODEL PREDICTING UPDRS BY MEAN TRACER UPTAKE IN BOTH SIDES OF PUTAMEN**							
	Constant	14.605	3.159		4.623	0.000	
1	Duration of PD (months)	1.096	0.533	0.340	2.054	0.051	
	Absorption level in putamen (ipsi-and contralateral)	−2.087	1.235	−0.263	−1.690	0.104	0.32 (*p* = 0.008)
2	rs6356 (carriership of A allele)	−4.571	1.842	−0.406	−2.481	0.020	0.46 (*p* = 0.002)

## References

[B1] Agid Y. (1991). Parkinson's disease: pathophysiology. Lancet 337, 1321–1324 10.1016/0140-6736(91)92989-F 1674304

[B2] Appel-Cresswell S. de la Fuente-Fernandez R. Galley S. McKeown M. J. (2010). Imaging of compensatory mechanisms in Parkinson's disease. Curr. Opin. Neurol. 23, 407–412 10.1097/WCO.0b013e32833b6019 20610991

[B3] Asselin M. C. Soghomonian J. J. Côté P. Y. Parent A. (1994). Striatal changes in preproenkephalin mRNA levels in parkinsonian monkeys. Neuroreport 5, 2137–2140 7865763 10.1097/00001756-199410270-00037

[B4] Barrett J. C. Fry B. Maller J. Daly M. J. (2005). Haploview: analysis and visualization of LD and haplotype maps. Bioinformatics 1, 263–265 10.1093/bioinformatics/bth457 15297300

[B5] Bernheimer H. Birkmayer W. Hornykiewicz O. Jellinger K. Seitelberger F. (1973). Brain dopamine and the syndromes of Parkinson and Huntington. Clinical, morphological and neurochemical correlations. J. Neurol. Sci. 20, 415–455 4272516 10.1016/0022-510x(73)90175-5

[B6] Bezard E. Crossman A. R. Gross C. E. Brotchie J. M. (2001a). Structures outside the basal ganglia may compensate for dopamine loss in the pre-symptomatic stages of Parkinson's disease. FASEB J. 15, 1092–1094 10.1096/fj.00-0637fje 11292678

[B7] Bezard E. Dovero S. Prunier C. Ravenscroft P. Chalon S. Guilloteau D. (2001b). Relationship between the appearance of symptoms and the level of nigrostriatal degeneration in a progressive 1-methyl-4-phenyl-1,2,3,6-tetrahydropyridine-lesioned macaque model of Parkinson's disease. J. Neurosci. 21, 6853–6861 11517273 10.1523/JNEUROSCI.21-17-06853.2001 PMC6763089

[B8] Bezard E. Ravenscroft P. Gross C. E. Crossman A. R. Brotchie J. M. (2001c). Upregulation of striatal preproenkephalin gene expression occurs before the appearance of Parkinsonian signs in 1-methyl-4-phenyl-1,2,3,6-tetrahydropyridine monkeys. Neurobiol. Dis. 8, 343–350 10.1006/nbdi.2000.0375 11300729

[B9] Bezard E. Gross C. E. Brotchie J. M. (2003). Presymptomatic compensation in Parkinson's disease is not dopamine-mediated. Trends Neurosci. 26, 215–221 12689773 10.1016/S0166-2236(03)00038-9

[B10] Biju G. de la Fuente-Fernández R. (2009). Dopaminergic function and progression of Parkinson's disease: PET findings. Parkinsonism Relat. Disord. 15 Suppl. 4, S38–S40 10.1016/S1353-8020(09)70833-8 20123555

[B11] Black K. J. Koller J. M. Campbell M. C. Gusnard D. A. Bandak S. I. (2010). Quantification of indirect pathway inhibition by the adenosine A2a antagonist SYN115 in Parkinson disease. J. Neurosci. 30, 16284–16292 10.1523/JNEUROSCI.2590-10.2010 21123574 PMC3008651

[B12] Braak H. Del Tredici K. Rub U. de Vos R. A. Jansen Steur E. N. Braak E. (2003). Staging of brain pathology related to sporadic Parkinson's disease. Neurobiol. Aging 24, 197–211 12498954 10.1016/s0197-4580(02)00065-9

[B13] Brotchie J. Fitzer-Attas C. (2009). Mechanisms compensating for dopamine loss in early Parkinson disease. Neurology 72 7 Suppl. S32–S38 10.1212/WNL.0b013e318198e0e9 19221312

[B14] Cerasa A. Hagberg G. E. Peppe A. Bianciardi M. Gioia M. C. Costa A. (2006). Functional changes in the activity of cerebellum and frontostriatal regions during externally and internally timed movement in Parkinson's disease. Brain Res. Bull. 71, 259–269 10.1016/j.brainresbull.2006.09.014 17113955

[B15] Chen C. C. Shih Y. Y. Chang C. (2012). Dopaminergic imaging of nonmotor manifestations in a rat model of Parkinson's disease by fMRI. Neurobiol. Dis. 49C, 99–106 10.1016/j.nbd.2012.07.020 22842018

[B16] Choi J. K. Chen Y. I. Hamel E. Jenkins B. G. (2006). Brain hemodynamic changes mediated by dopamine receptors: role of the cerebral microvasculature in dopamine-mediated neurovascular coupling. Neuroimage 30, 700–712 10.1016/j.neuroimage.2005.10.029 16459104

[B17] Creese I. Burt D. R. Snyder S. H. (1977). Dopamine receptor binding: enhancement accompanying lesion-induced behavioural supersensitivity. Science 197, 596–598 10.1126/science.877576 877576

[B18] Dacko S. Schneider J. S. (1991). Metenkephalin immunoreactivity in the basal ganglia in symptomatic and asymptomatic MPTP-exposed monkeys: correlation with degree of parkinsonian symptoms. Neurosci. Lett. 127, 49–52 10.1016/0304-3940(91)90892-W 1881617

[B19] Dauer W. Przedborski S. (2003). Parkinson's disease: mechanisms and models. Neuron 39, 889–909 10.1016/S0896-6273(03)00568-3 12971891

[B20] de la Fuente-Fernández R. Schulzer M. Kuramoto L. Cragg J. Ramachandiran N. Au W. L. (2011). Age-specific progression of nigrostriatal dysfunction in Parkinson's disease. Ann. Neurol. 69, 803–810 10.1002/ana.22284 21246604

[B21] Du X. Iacovitti L. (1995). Synergy between growth factors and transmitters required for catecholamine differentiation in brain neurons. J. Neurosci. 15, 5420–5427 7542701 10.1523/JNEUROSCI.15-07-05420.1995 PMC6577854

[B22] Elsworth J. D. Taylor J. R. Sladek J. R. Jr. Collier T. J. Redmond D. E. Jr. Roth R. H. (2000). Striatal dopaminergic correlates of stable Parkinsonism and degree of recovery in old-world primates one year after MPTP treatment. Neuroscience 95, 399–408 10.1016/S0306-4522(99)00437-6 10658619

[B23] Falardeau P. Bedard P. J. Di Paolo T. (1988). Relation between brain dopamine loss and D2 dopamine receptor density in MPTP monkeys. Neurosci. Lett. 86, 225–229 10.1016/0304-3940(88)90575-7 2966905

[B24] Gubellini P. Picconi B. Bari M. Battista N. Calabresi P. Centonze D. (2002). Experimental parkinsonism alters endocannabinoid degradation: implications for striatal glutamatergic transmission. J. Neurosci. 22, 6900–6907 12177188 10.1523/JNEUROSCI.22-16-06900.2002 PMC6757864

[B25] Guigoni C. Doudnikoff E. Li Q. Bloch B. Bezard E. (2007). Altered D(1) dopamine receptor trafficking in parkinsonian and dyskinetic non-human primates. Neurobiol. Dis. 26, 452–463 10.1016/j.nbd.2007.02.001 17350277

[B26] Hefti F. Enz A. Melamed E. (1985). Partial lesions of the nigrostriatal pathway in the rat. Acceleration of transmitter synthesis and release of surviving dopaminergic neurons by drugs. Neuropharmacology 24, 19–23 2858830 10.1016/0028-3908(85)90090-5

[B27] Herrero M. T. Augood S. J. Hirsch E. C. Javoy-Agid F. Luquin M. R. Agid Y. (1995). Effects of L-DOPA on preproenkephalin and preprotachykinin gene expression in the MPTP-treated monkey striatum. Neuroscience 68, 1189–1198 10.1016/0306-4522(95)00120-8 8544992

[B28] Huot P. Lévesque M. Parent A. (2007). The fate of striatal dopaminergic neurons in Parkinson's disease and Huntington's chorea. Brain 130(Pt 1), 222–232 10.1093/brain/awl332 17142832

[B29] Hurley M. J. Mash D. C. Jenner P. (2001). Dopamine D(1) receptor expression in human basal ganglia and changes in Parkinson's disease. Brain Res. Mol. Brain Res. 87, 271–279 10.1016/S0169-328X(01)00022-5 11245931

[B30] Javoy-Agid F. Hirsch E. C. Dumas S. Duyckaerts C. Mallet J. Agid Y. (1990). Decreased tyrosine hydroxylase messenger RNA in the surviving dopamine neurons of the substantia nigra in Parkinson's disease: an *in situ* hybridization study. Neuroscience 38, 245–253 10.1016/0306-4522(90)90389-L 1979431

[B31] Jenner P. (2008). Molecular mechanisms of L-DOPA-induced dyskinesia. Nat. Rev. Neurosci. 9, 665–677 10.1038/nrn2471 18714325

[B32] Johnson M. D. Miocinovic S. McIntyre C. C. Vitek J. L. (2008). Mechanisms and targets of deep brain stimulation in movement disorders. Neurotherapeutics 5, 294–308 10.1016/j.nurt.2008.01.010 18394571 PMC2517242

[B33] Kojovic M. Bologna M. Kassavetis P. Murase N. Palomar F. J. Berardelli A. (2012). Functional reorganization of sensorimotor cortex in early Parkinson disease. Neurology 78, 1441–1448 10.1212/WNL.0b013e318253d5dd 22517098 PMC3345788

[B34] Kuriakose R. Stoessl A. J. (2010). Imaging the nigrostriatal system to monitor disease progression and treatment-induced complications. Prog. Brain Res. 184, 177–192 10.1016/S0079-6123(10)84009-9 20887875

[B35] Lee C. S. Samii A. Sossi V. Ruth T. J. Schulzer M. Holden J. E. (2000). *In vivo* positron emission tomographic evidence for compensatory changes in presynaptic dopaminergic nerve terminals in Parkinson's disease. Ann. Neurol. 47, 493–503 10762161

[B36] Lee T. Seeman P. Rajput A. Farley I. J. Hornykiewicz O. (1978). Receptor basis for dopaminergic supersensitivity in Parkinson's disease. Nature 273, 150–151 692671 10.1038/273059a0

[B37] Martinu K. Monchi O. (2012). Cortico-basal ganglia and cortico-cerebellar circuits in Parkinson's disease: pathophysiology or compensation? Behav. Neurosci. 10.1037/a0031226 [Epub ahead of print]. 23244290

[B38] Melamed E. Hefti F. Wurtman R. J. (1982). Compensatory mechanisms in nigrostriatal dopaminergic neurons in parkinsonism. Studies in an animal model. Isr. J. Med. Sci. 18, 159–163 10.1037/a0031226 6121770

[B39] Nandhagopal R. Kuramoto L. Schulzer M. Mak E. Cragg J. McKenzie J. (2011). Longitudinal evolution of compensatory changes in striatal dopamine processing in Parkinson's disease. Brain 134(Pt 11), 3290–3298 10.1093/brain/awr233 22075521

[B40] Obeso J. A. Rodriguez-Oroz M. C. Goetz C. G. Marin C. Kordower J. H. Rodriguez M. (2010). Missing pieces in the Parkinson's disease puzzle. Nat. Med. 16, 653–661 10.1038/nm.2165 20495568

[B41] Obeso J. A. Rodriguez-Oroz M. C. Lanciego J. L. Rodriguez Diaz M. (2004). How does Parkinson's disease begin? The role of compensatory mechanisms. Trends Neurosci. 27, 125–127 10.1016/j.tins.2003.12.006 15036875

[B42] Obeso J. A. Schapira A. H. (2009). Compensatory mechanisms in Parkinson's disease. Mov. Disord. 24, 153–154 18649387 10.1002/mds.22177

[B43] Palmer S. J. Li J. Wang Z. J. McKeown M. J. (2010). Joint amplitude and connectivity compensatory mechanisms in Parkinson's disease. Neuroscience 166, 1110–1118 10.1016/j.neuroscience.2010.01.012 20074617

[B44] Palmer S. J. Ng B. Abugharbieh R. Eigenraam L. McKeown M. J. (2009). Motor reserve and novel area recruitment: amplitude and spatial characteristics of compensation in Parkinson's disease. Eur. J. Neurosci. 29, 2187–2196 10.1111/j.1460-9568.2009.06753.x 19490021

[B45] Perez X. A. Parameswaran N. Huang L. Z. O'Leary K. T. Quik M. (2008). Pre-synaptic dopaminergic compensation after moderate nigrostriatal damage in non-human primates. J. Neurochem. 105, 1861–1872 10.1111/j.1471-4159.2008.05268.x 18248617 PMC3264543

[B46] Pisani A. Fezza F. Galati S. Battista N. Napolitano S. Finazzi-Agrò A. (2005). High endogenous cannabinoid levels in the cerebrospinal fluid of untreated Parkinson's disease patients. Ann. Neurol. 57, 777–779 10.1002/ana.20462 15852389

[B47] Porritt M. J. Batchelor P. E. Hughes A. J. Kalnins R. Donnan G. A. Howells D. W. (2000). Newdopaminergic neurons in Parkinson's disease striatum. Lancet 356, 44–45 10.1016/S0140-6736(00)02437-5 10892768

[B48] Prediger R. D. (2010). Effects of caffeine in Parkinson's disease: from neuroprotection to the management of motor and non-motor symptoms. J. Alzheimers Dis. 20 Suppl. 1, S205–S220 10.3233/JAD-2010-091459 20182024

[B49] Quik M. Bordia T. O'Leary K. (2007). Nicotinic receptors as CNS targets for Parkinson's disease. Biochem. Pharmacol. 74, 1224–1234 10.1016/j.bcp.2007.06.015 17631864 PMC2046219

[B50] Rascol O. Sabatini U. Brefel C. Fabre N. Rai S. Senard J. M. (1998). Cortical motor overactivation in parkinsonian patients with L-dopa-induced peak-dose dyskinesia. Brain 121(Pt 3), 527–533 10.1093/brain/121.3.527 9549528

[B51] Rascol O. Sabatini U. Chollet F. Celsis P. Montastruc J. L. Marc-Vergnes J. P. (1992). Supplementary and primary sensory motor area activity in Parkinson's disease. Regional cerebral blood flow changes during finger movements and effects of apomorphine. Arch. Neurol. 49, 144–148 10.1001/archneur.1992.00530260044017 1736846

[B52] Rosenblad C. Martinez-Serrano A. Björklund A. (1998). Intrastriatal glial cell line-derived neurotrophic factor promotes sprouting of spared nigrostriatal dopaminergic afferents and induces recovery of function in a rat model of Parkinson's disease. Neuroscience 82, 129–137 10.1016/S0306-4522(97)00269-8 9483509

[B53] Sánchez-Pernaute R. Jenkins B. G. Choi J. K. Iris Chen Y. C. Isacson O. (2007). *In vivo* evidence of D3 dopamine receptor sensitization in parkinsonian primates and rodents with l-DOPA-induced dyskinesias. Neurobiol. Dis. 27, 220–227 10.1016/j.nbd.2007.04.016 17588764 PMC2674779

[B54] Shinotoh H. Inoue O. Hirayama K. Aotsuka A. Asahina M. Suhara T. (1993). Dopamine D1 receptors in Parkinson's disease and striatonigral degeneration: a positron emission tomography study. J. Neurol. Neurosurg. Psychiatr. 56, 467–472 8505636 10.1136/jnnp.56.5.467 PMC1015002

[B55a] Sossi V. de la Fuente-Fernández R. Schulzer M. Troiano A. R. Ruth T. J. Stoessl A. J. (2007). Dopamine transporter relation to dopamine turnover in Parkinson's disease: a positron emission tomography study. Ann. Neurol. 62, 468–474 10.1002/ana.21204 17886297

[B55] Stoessl A. J. Martin W. W. McKeown M. J. Sossi V. (2011). Advances in imaging in Parkinson's disease. Lancet Neurol. 10, 987–1001 10.1016/S1474-4422(11)70214-9 22014434

[B56] Sugita M. Izuno T. Tatemichi M. Otahara Y. (2001). Meta-analysis for epidemiologic studies on the relationship between smoking and Parkinson's disease. J. Epidemiol. 11, 87–94 10.2188/jea.11.87 11388498 PMC11638025

[B57] Tandé D. Höglinger G. Debeir T. Freundlieb N. Hirsch E. C. François C. (2006). New striatal dopamine neurons in MPTP-treated macaques results from a phenotypic shift and not neurogenesis. Brain 129, 1194–1200 10.1093/brain/awl041 16481374

[B58] Todd R. D. Carl J. Harmon S. O'Malley K. L. Perlmutter J. S. (1996). Dynamic changes in striatal dopamine D2 and D3 receptor protein and mRNA in response to 1-methyl-4-phenyl-1,2,3,6-tetrahydropyridine (MPTP) denervation in baboons. J. Neurosci. 16, 7776–7782 8922433 10.1523/JNEUROSCI.16-23-07776.1996 PMC6579075

[B59] Tokuoka H. Muramatsu S. Sumi-Ichinose C. Sakane H. Kojima M. Aso Y. (2011). Compensatory regulation of dopamine after ablation of the tyrosine hydroxylase gene in the nigrostriatal projection. J. Biol. Chem. 286, 43549–43558 10.1074/jbc.M111.284729 22027820 PMC3234829

[B60] Uhl G. R. Walther D. Mash D. Faucheux B. Javoy-Agid F. (1994). Dopamine transporter messenger RNA in Parkinson's disease and control substantia nigra neurons. Ann. Neurol. 35, 494–498 10.1002/ana.410350421 8154880

[B61] van der Stelt M. Fox S. H. Hill M. Crossman A. R. Petrosino S. Di Marzo V. (2005). A role for endocannabinoids in the generation of parkinsonism and levodopa-induced dyskinesia in MPTP-lesioned non-human primate models of Parkinson's disease. FASEB J. 19, 1140–1142 10.1096/fj.04-3010fje 15894565

[B62] van Nuenen B. F. Helmich R. C. Buenen N. van de Warrenburg B. P. Bloem B. R. Toni I. (2012). Compensatory activity in the extrastriate body area of Parkinson's disease patients. J. Neurosci. 32, 9546–9553 10.1523/JNEUROSCI.0335-12.2012 22787040 PMC6622256

[B63] van Nuenen B. F. van Eimeren T. van der Vegt J. P. Buhmann C. Klein C. Bloem B. R. (2009). Mapping preclinical compensation in Parkinson's disease: an imaging genomics approach. Mov. Disord. 24 Suppl. 2, S703–S710 10.1002/mds.22635 19877238

[B64] Vila M. Périer C. Féger J. Yelnik J. Faucheux B. Ruberg M. (2000). Evolution of changes in neuronal activity in the subthalamic nucleus of rats with unilateral lesion of the substantia nigra assessed by metabolic and electrophysiological measurements. Eur. J. Neurosci. 12, 337–344 10.1046/j.1460-9568.2000.00901.x 10651888

[B65] Weinreb O. Amit T. Bar-Am O. Youdim M. B. (2007). Induction of neurotrophic factors GDNF and BDNF associated with the mechanism of neurorescue action of rasagiline and ladostigil: new insights and implications for therapy. Ann. N.Y. Acad. Sci. 1122, 155–168 10.1196/annals.1403.011 18077571

[B66] Yu H. Sternad D. Corcos D. M. Vaillancourt D. E. (2007). Role of hyperactive cerebellum and motor cortex in Parkinson's disease. Neuroimage 35, 222–233 10.1016/j.neuroimage.2006.11.047 17223579 PMC1853309

[B67] Zigmond M. J. Abercrombie E. D. Berger T. W. Grace A. A. Stricker E. M. (1990). Compensations after lesions of central dopaminergic neurons: some clinical and basic implications. Trends Neurosci. 13, 290–296 1695406 10.1016/0166-2236(90)90112-n

[B67a] Zigmond M. J. Acheson A. L. Stachowiak M. K. Stricker E. M. (1984). Neurochemical compensation after nigrostriatal bundle injury in an animal model of preclinical parkinsonism. Arch. Neurol. 41, 856–861 10.1001/archneur.1984.04050190062015 6147127

